# Identification of Ultra-Short Laser Parameters for a 3D Model of a Thin Metal Film Using the Lattice Boltzmann Method

**DOI:** 10.3390/ma18225079

**Published:** 2025-11-07

**Authors:** Adam Długosz, Anna Korczak

**Affiliations:** Department of Computational Mechanics and Engineering, Faculty of Mechanical Engineering, Silesian University of Technology, Konarskiego 18A, 44-100 Gliwice, Poland; anna.korczak@polsl.pl

**Keywords:** heat transfer, ultra-short laser pulse, lattice Boltzmann method, inverse problem, identification, metaheuristic algorithms

## Abstract

The paper uses the model of the coupled Boltzmann transport equations, numerically modeled using the lattice Boltzmann method, which describes the impact of an ultra-short laser on thin metal surfaces. The main reason for using this method is the need to create an appropriate model on a very small scale, both in terms of space and time. In the present study, a three-dimensional model is used, for which, in the case of optimization or identification tasks, the characteristics of the obtained numerical solution are quite different from those of other numerical methods and models. The proposed and numerically implemented task of identifying parameters characterizing the laser pulse, such as the power of the stationary laser source, the laser-beam absorptivity coefficient, and the radius of the laser beam, appropriately illustrates the problem. The problem has also been solved for ideal deterministic (no noise) and randomly disturbed (with noise) values of measured temperatures. Three different optimization algorithms are used to solve the inverse task. Several variants of the identification tasks, differing in terms of the number of temperature measurement points, are solved. The results and effectiveness of the identification tasks are compared for the best solution, as well as the statistical measures.

## 1. Introduction

Heat transfer in thin metal films exposed to ultra-short laser pulses can manifest in various technical systems and, nowadays, is very deeply investigated using experimental methods [1,2,3] as well as numerical simulations [4,5,6]. Given the rapid nature of this process, it is important to model such problems using appropriate mathematical models and numerical techniques [7]. The standard thermal conductivity models in such a case are obviously inadequate. One possible extension of the standard approach is a generalization of Fourier’s law that can introduce a relaxation time between the heat flux and the temperature gradient, e.g., the Cattaneo–Vernotte equation [8]. Another option for modeling the thermal processes in microdomains subjected to strong external thermal effects is the application of the dual phase lag equation (DPLE). Such an approach is distinguished by the two lag times—the relaxation time of the heat flux vector and the relaxation time of the temperature gradient. Different variants of the method, which include the second-order time derivative and the higher-order mixed derivative in time and space, can be modeled [9,10,11].

DPLE can be successfully used not only for metallic materials [12,13] but also in modeling bioheat flow [14,15,16]. Additionally, from the aforementioned methods, heat flow in metals is analyzed by considering two types of energy carriers, i.e., electrons and phonons [17]. This entails solving a system of two coupled Boltzmann transport equations (BTE) [18]. The phonon BTE has been extensively employed to forecast phonon transport in the subcontinuum regime, where the wave characteristics of energy carriers (i.e., electrons in metals, phonons in semiconductors, and dielectrics) can be disregarded. The lattice Boltzmann method (LBM), widely utilized for fluid and thermal transport issues, offers an efficient numerical approach for solving the BTE. Moreover, it can be expanded to address electron transport and coupled phonon-electron challenges.

In the literature, many applications of BTE to solve heat flow problems can be found, including coupled ones. However, most analyses are carried out in relation to one- or two-dimensional problems. One example is a 2D model in which the heat source forms a molten pool; a method to investigate the heat transfer inside the pool has been proposed by Shen and Zhang [19,20]. The authors, in order to better align with practical physics, investigated solid–liquid phase changes considering temperature-dependent thermal properties. Therefore, this study introduces a double distribution lattice Boltzmann model for annular heat sources, accounting for variations in thermal conductivity and thermal diffusivity with temperature. The study examines the flow patterns of a molten pool irradiated by an annular laser, considering the combined effects of natural convection and Marangoni convection. Moreover, the annular heat source offers distinct controllable parameters, including inner and outer annular radii and the hollow ratio.

Another group of papers uses only phonon BTE to describe some phenomena in semiconductors. Saurav and Mazumder studied the thermal transport of interface structures based on a silicon substrate in cylindrical coordinates [21]. They investigated the phase difference between the pump and probe laser signals that were calculated for various pump laser modulation frequencies. Results were derived, with and without considering optical phonons, using two different relaxation timescale expressions for scattering taken from the literature. It was observed that incorporating optical phonons notably enhanced the consistency between the measured and calculated phase differences. Hu et al. analyzed interfacial thermal transport across a GaN/AlN interface, considering the diffuse mismatch model, the maximum transmission model, the higher harmonic inelastic model, and the modified higher harmonic inelastic model to analyze their influence on the results [22]. Their findings indicate that incorporating inelastic scattering boosts the heat flux contribution from low-frequency phonons, alters the phonon distribution close to the interface, and amplifies phonon nonequilibrium. This underscores the significance of inelastic interfacial scattering in simulations. Upon examining the phonon distribution, it is evident that temperature and thickness impact the interfacial heat transport by reshuffling phonons.

The BTE can also be adopted, as it can cover the ballistic to diffusive regime, to investigate the size effect on thermal constriction resistance. In research by Zahiri et al., by incorporating phonon dispersion and polarization, the phonon BTE is used to reveal details of thermal transport through a constriction in silicon [23]. The numerical method applies across various Knudsen numbers and constriction ratios. Results from gray, two-band, and five-band nongray BTE simulations for different constriction widths are contrasted. Under the gray assumption, they observed a more diffusive scenario with identical geometry, which leads to reduced thermal resistance.

**Figure 1:**
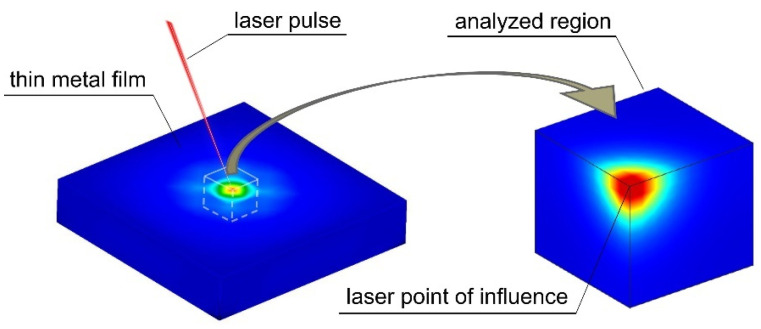
Three-dimensional model of a thin metal film exposed to an ultra-short laser pulse.

The present paper adopts an LBM to solve the coupled BTE in the formulated task of identifying selected parameters of a laser interacting with a thin metal layer. Unlike the research mentioned above, the model considered in this paper is a three-dimensional model. The proposed approach is a substantial extension of earlier work on this issue [24]. In the present paper, the body (represented as a 3D cubic model) is irradiated by a pulse and modeled as a semispherical Gaussian distribution of energy density (Figure 1). The distribution of temperature in space and time is then numerically calculated using LBM.

As shown in Figure 1, symmetry conditions were applied that made it possible to reduce the model and, thus, the number of degrees of freedom of the analyzed system. This study addresses the inverse problem of determining parameters that characterize the laser pulse: the power of the stationary laser source and the laser-beam absorptivity coefficient. The identification process is performed numerically using a formulated identification function, which relies on a norm of the difference between the current and measured temperature values at specified measurement points. In the past, inverse problems involving laser–material interaction were mainly investigated using macroscopic heat conduction models or continuum-level approximations based on Finite Element Method and Finite Difference Method techniques. With the development of the Lattice Boltzmann framework, it became possible to capture microscale effects, but applications to inverse identification tasks have remained limited.

The phenomenon of laser interaction on the microscale, in which the geometric model has dimensions on the order of a few hundred nanometers and the time of laser interaction is measured in picoseconds, makes it impossible to conduct experimental studies. Measured temperature values in this research are understood to be simulated numerically as a known output of the considered system. Typically, inverse tasks belong to the ill-posed problems [25], although they may be ill-conditioned even when the problem is well-posed, which makes it necessary to attach great importance to the methods and algorithms used to solve the tasks of identification. Identification in physical systems, including thermal issues, especially when the value of the objective function is calculated from the results of numerical simulations using different methods, can cause ambiguities and multimodality.

Ambiguity in this type of task can be reduced by, for example, measuring quantities from other physical fields or increasing the number of measurement points, although this is not always applicable. On the other hand, the multimodality of the objective function requires the need for appropriate identification algorithms. For algorithms that require determination of the starting point of the optimization, whether gradient or non-gradient, it becomes necessary to search for a solution for different starting points [26]. The use of regularization of the objective function can help reduce its degree of multimodality. However, this process requires a cautious approach because its inappropriate application may separate us from the real problem of identification.

In contrast to the group of classical optimization methods, another approach free from the above disadvantage is the use of one of the global optimization methods. Suitable methods from this group (e.g., an evolutionary algorithm), which process not one but a set of solutions in each iteration, help ensure the correct balance between exploration and exploitation of the solution space [27,28,29]. Nowadays, artificial neural networks with appropriate topology are also often used to solve such tasks [30,31,32]. Another major problem in identification tasks is measurement uncertainties, which can make it significantly more difficult to find the correct solution [33]. In the present work, three different optimization algorithms were used to solve the identification task for the problem presented above—the evolutionary algorithm (EA), the Monte Carlo method, and the Nelder-Mead algorithm—for the differently assumed starting points of the identification procedure.

The Nelder–Mead algorithm works comparable to a lot of different classical gradient methods, where searching is utilized along the direction of improvement of the objective function, but it doesn’t need fitness function gradient because it uses a modified simplex method. This makes it a good candidate for use in the identification problem under consideration, because determining the gradient of the identification functional with respect to the variable target parameters would be very problematic. It works very fast and efficiently, but for multimodal objective functions there is a high probability of finding a local solution. For this reason, this type of algorithm should be run multiple times for different starting points. For the Monte Carlo optimization methods search of the solution space is random. These algorithms do not use information from previous iterations of their operation, but with a sufficiently large number of random trials, they can be valuable in solving problems with multiple local optima and complex constraints. Evolutionary algorithms, which are a floating-point version of the classic genetic algorithm with modified evolutionary operators, are an excellent representative of metaheuristic optimization methods. The group of metaheuristic optimization methods currently includes dozens of different algorithms that have various biological inspirations, but share common features like: exchange of information between individuals (crossover), element of randomness (mutation), selection of better adapted individuals in subsequent generations (selection).

Each of these three optimization methods selected and applied in this work has completely different properties in terms of its ability to search for admissible non-trivial solution spaces, which justifies their selection as representative for verifying the effectiveness of the task of identifying the parameters of an ultrashort laser on a three-dimensional model of a thin metal layer using the LBM.

The novelty of the present work lies in extending the LBM formulation to a fully three-dimensional coupled electron–phonon system under ultra-short laser irradiation, combined with an optimization scheme for parameter identification. This integrated approach enables the analysis of processes that are currently beyond experimental verification, offering a new computational framework for nanoscale heat transfer inverse problems.

The present paper is organized as follows. Section 2 presents a formulation of the heat flow in metals on the microscale by means of the BTE and LBM. This section also includes the formulation of the identification function with a description of the considered identification variants. Section 3 contains a diagram of the identification procedure along with descriptions of the optimization algorithms employed. The obtained results for the identification tasks are included in Section 4, while a discussion on them is presented in Section 5. The paper concludes, in Section 6, with an analysis of the conducted research.

## 2. Formulation of the Problem

### 2.1. Heat Flow in Metals by Means of the Boltzmann Transport Equations

When analyzing heat flow in metals on the microscale, we can consider two types of energy carriers—electrons and phonons—leading to a solution of a system of two coupled Boltzmann transport equations, i.e., [34].

(1) $$\left\{\begin{array}{l} \frac{\partial f_{e}}{\partial t}+v_{e}\cdot \nabla f_{e}=\frac{f_{e}^{0}-f_{e}}{\tau_{e}}+g_{e}\\ \frac{\partial f_{ph}}{\partial t}+v_{ph}\cdot \nabla f_{ph}=\frac{f_{ph}^{0}-f_{ph}}{\tau_{ph}}+g_{ph} \end{array}\right.$$

where $f_{e}$ and $f_{\mathrm{ph}}$ are the distribution functions of the electrons and phonons, respectively, and $f_{e}^{0}$ and $f_{ph}^{0}$ are the distribution functions of the carriers at the equilibrium state defined by the Bose-Einstein statistic for the phonons and the Fermi-Dirac statistic for the electrons, respectively. Moreover, $v_{e}$ and $v_{ph}$ [nm/ps] are the velocity vectors of the carrier group, $\tau_{e}$ and $\tau_{ph}$ [ps] are the relaxation times of the carriers, and $g_{e}$ and $g_{ph}$ [1/ps] are the carrier generation coefficients consistent with the “scattering” of the electron-phonon system.

The given system of equations can be transformed into an equivalent form with respect to the energy density of the carriers, so that [35]

(2) $$\left\{\begin{array}{l} \frac{\partial e_{e}}{\partial t}+v_{e}\cdot \nabla e_{e}=\frac{e_{e}^{0}-e_{e}}{\tau_{e}}+Q_{e}\\ \frac{\partial e_{ph}}{\partial t}+v_{ph}\cdot \nabla e_{ph}=\frac{e_{ph}^{0}-e_{ph}}{\tau_{ph}}+Q_{ph} \end{array}\right.$$

where $e_{e}$ and $e_{ph}$ [J/m^3^] are the energy densities of the carriers, $e_{e}^{0}$ and $e_{ph}^{0}$ [J/m^3^] are the energy densities of the carriers at equilibrium, and $Q_{e}$ and $Q_{ph}$ [W/m^3^] are the functions of the source.

The energy densities of the electrons and phonons as a function of temperature are calculated from the following formulae [36]:

(3) $$e_{e}(T_{e})=\left(n_{e}\frac{\pi^{2}}{2}\frac{k_{b}^{2}}{\varepsilon_{F}}\right)T_{e}^{2}$$

(4) $$e_{ph}(T_{ph})=\left(\frac{9\eta k_{b}}{\Theta_{D}^{3}}\;\int_{0}^{\Theta_{D}/T_{ph}}\frac{z^{3}}{\exp(z)-1}dz\right)T_{ph}^{4}$$

where $T_{e}$ and $T_{ph}$ [K] are the temperatures of the carriers, *k_b_* is the Boltzmann constant, $\varepsilon_{F}$ is the Fermi energy, $n_{e}$ [1/m^3^] is the electron density, Θ_D_ [K] is the Debye temperature, and η [1/m^3^] is the phonon density. The latter is explicitly expressed as follows [36]:

(5) $$\eta =\frac{1}{6\pi^{2}}{\left(\frac{k_{b}\Theta_{D}}{ћ\omega }\right)}^{3}$$

The source components per unit volume of the electrons and phonons are expressed via

(6) $$Q_{e}=Q^{\prime }-G(T_{e}-T_{ph})$$

(7) $$Q_{ph}=G(T_{e}-T_{ph})$$

respectively, where $Q^{\prime }$ is the heat source component associated with laser irradiation and *G* is the coupling coefficient that characterizes the energy exchange between electrons and phonons.

A three-dimensional Cartesian coordinate system for the transient temperature field in metals can be described by a system of Boltzmann transport equations, which have the following form:

(8) $$\left\{\begin{array}{l} \frac{\partial {\bar{e}}_{e}}{\partial t}+v_{e\;x}\frac{\partial {\bar{e}}_{e}}{\partial x}+v_{e\;y}\frac{\partial {\bar{e}}_{e}}{\partial y}+v_{e\;z}\frac{\partial {\bar{e}}_{e}}{\partial z}=-\frac{{\bar{e}}_{e}-{\bar{e}}_{e}^{0}}{{\bar{\tau }}_{r}}+Q_{e}\\ \frac{\partial {\bar{e}}_{ph}}{\partial t}+v_{ph\;x}\frac{\partial {\bar{e}}_{ph}}{\partial x}+v_{ph\;y}\frac{\partial {\bar{e}}_{ph}}{\partial y}+v_{ph\;z}\frac{\partial {\bar{e}}_{ph}}{\partial z}=-\frac{{\bar{e}}_{ph}-{\bar{e}}_{ph}^{0}}{{\bar{\tau }}_{r}}+Q_{ph} \end{array}\right.$$

**Figure 2:**
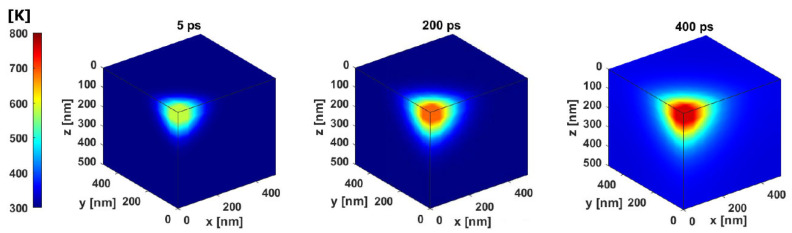
Distribution of the temperature in the model for consecutive time intervals.

A numerical implementation of the problem is realized by means of the so-called D3Q7 model [37]. More details about such numerical implementation are included in Section 2.2. The developed model allows for the solving of direct heat transfer in the case of ultra-thin metal layers exposed to the laser beam. Due to symmetry considerations, only a quarter of the model was analyzed. In this model, adiabatic boundary conditions were applied to all sides of the cube, whereas the heat source was only applied to one of the corners. Examples of the distribution of the temperature in the model in such an extremely small spatial-temporal regime are presented in Figure 2.

### 2.2. Implementation of the Identification Problem

**Figure 3:**
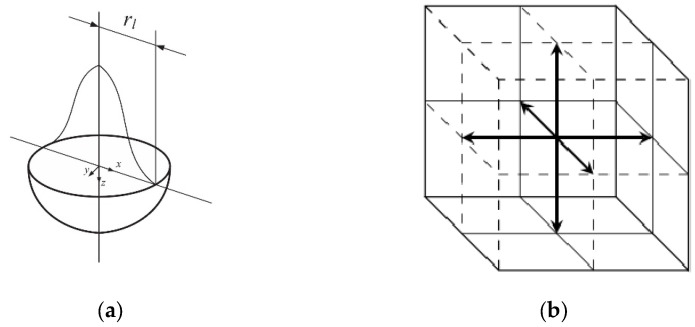
(**a**) Semispherical shape model of the heat source [37] and (**b**) directions of the D3Q7 model [38].

When considering the impact of the laser on the material in the numerical model, the appropriate shape of the heat source in 3D space must be considered. Many geometric models have been developed so far, the following of which are worth mentioning: cylindrical, semispherical, semi-ellipsoidal, and conical shapes. This paper adopts one of the more popular models, i.e., semispherical, which is described in more detail elsewhere [38]. Figure 3a presents the applied model of the heat source. For this case, heat source $Q^{\prime }$ in Equation (6), modeled in a Cartesian coordinate system, is described by the following formula:

(9) $$\;Q^{\prime }(x,y,z)=\frac{2^{5/2}\beta P}{\pi^{3/2}r_{l}^{3}}\exp\left[-2\frac{x^{2}+y^{2}+z^{2}}{r_{l}^{2}}\right]$$

where *P* is the power of the stationary laser pulse, β is the laser beam absorptivity, and *r_l_* is the radius of the laser beam.

The aforementioned laser parameters are identified in the present work based on the temperatures in the sensor points and the use of the identification function (10), which is defined and described below. The multiplication of variables β and *P* could be treated as one separate parameter. Therefore, it was decided not to identify all three parameters at the same time, but in the form of two variants of identification. The two variants of identification tasks were considered due to the form of the numerator in the Formula (9), i.e.,

- Variant 1: simultaneous identification of *r_l_* and β
- Variant 2: simultaneous identification of *r_l_* and *P*.

The identification problem of such parameters was solved by minimizing the following function:

(10) $$\underset{x}{\min}\;\;\;J\left(x\right)=\sum_{i=1}^{n}\sum_{i=1}^{m}{\left[T_{ij}\left(x\right)-{\hat{T}}_{ij}\left(x\right)\right]}^{2}$$

where *n* is the number of sensor points, *m* is the number of time intervals, **x** is a vector of identification parameters $T_{ij}$ that are computed (simulated numerically) values of the temperature in the sensors, and ${\hat{T}}_{ij}$ are “measured” (postulated) values of the temperature in the sensors.

As mentioned in Section 1, it is impossible to perform real experiments that determine temperatures in such an extremely small spatial-temporal regime. This means that the measured (postulated) temperatures are also determined by numerical simulations. This is carried out for known values of the identified parameters, which is a commonly used approach to solve the boundary-value problem. The temperature field in the microscale domain is calculated by means of a three-dimensional D3Q7 model using the lattice Boltzmann method.

In the analyzed three-dimensional model D3Q7, phonons and electrons are assumed to vibrate in seven directions (Figure 3b). The velocities of the electrons and phonons in the main directions of the lattice are a discrete set, which are given as follows:

(11) $$\begin{array}{l} v_{e0}=\left[0,\;0,\;0\right]\;,\;\;v_{e1}=\left[v_{e},0,\;0\right],\\ v_{e2}=\left[-v_{e},0,\;0\right]\;,\;\;v_{e3}=\left[0,v_{e},\;0\right],\\ v_{e4}=\left[0,-v_{e},\;0\right]\;,\;\;v_{e5}=\left[0,\;0,\;v_{e}\right],\\ \;\;\;v_{e6}=\left[0,\;0,\;-v_{e}\right], \end{array}$$

(12) $$\begin{array}{l} v_{ph0}=\left[0,0,\;0\right]\;,\;\;\;\;\;v_{ph1}=\left[v_{ph},0,\;0\right],\\ v_{ph2}=\left[-v_{ph},0,\;0\right]\;,\;\;v_{ph3}=\left[0,v_{ph},\;0\right],\\ v_{ph4}=\left[0,-v_{ph},\;0\right]\;,\;\;v_{ph5}=\left[0,\;0,\;v_{ph}\right],\\ \;\;\;\;v_{ph6}=\left[0,\;0,\;-v_{ph}\right]. \end{array}$$

Taking into account the above velocities and approximating the partial derivatives with the corresponding differential quotients, the system of equations then takes the form

(13) $$\left\{\begin{array}{l} {\left(e_{e0}\right)}_{i,j,k}^{f+1}=\left(1-\Delta t/\tau_{e}\right){\left(e_{e0}\right)}_{i,j,k}^{f}+\Delta t/\tau_{e}\cdot {\left(e_{e0}^{0}\right)}_{i,j,k}^{f}+\Delta tq_{v}\\ {\left(e_{e1}\right)}_{i+1,j,k}^{f+1}=\left(1-\Delta t/\tau_{e}\right){\left(e_{e1}\right)}_{i,j,k}^{f}+\Delta t/\tau_{e}\cdot {\left(e_{e1}^{0}\right)}_{i,j,k}^{f}+\Delta tq_{v}\\ {\left(e_{e2}\right)}_{i-1,j,k}^{f+1}=\left(1-\Delta t/\tau_{e}\right){\left(e_{e2}\right)}_{i,j,k}^{f}+\Delta t/\tau_{e}\cdot {\left(e_{e2}^{0}\right)}_{i,j,k}^{f}+\Delta tq_{v}\\ {\left(e_{e3}\right)}_{i,j+1,k}^{f+1}=\left(1-\Delta t/\tau_{e}\right){\left(e_{e3}\right)}_{i,j,k}^{f}+\Delta t/{\bar{\tau }}_{e}\cdot {\left(e_{e3}^{0}\right)}_{i,j,k}^{f}+\Delta tq_{v}\\ {\left(e_{e4}\right)}_{i,j-1,k}^{f+1}=\left(1-\Delta t/\tau_{e}\right){\left(e_{e4}\right)}_{i,j,k}^{f}+\Delta t/\tau_{e}\cdot {\left(e_{e4}^{0}\right)}_{i,j,k}^{f}+\Delta tq_{v}\\ {\left(e_{e5}\right)}_{i,j,k+1}^{f+1}=\left(1-\Delta t/\tau_{e}\right){\left(e_{e5}\right)}_{i,j,k}^{f}+\Delta t/\tau_{e}\cdot {\left(e_{e5}^{0}\right)}_{i,j,k}^{f}+\Delta tq_{v}\\ {\left(e_{e6}\right)}_{i,j,k-1}^{f+1}=\left(1-\Delta t/\tau_{e}\right){\left(e_{e6}\right)}_{i,j,k}^{f}+\Delta t/\tau_{e}\cdot {\left(e_{e6}^{0}\right)}_{i,j,k}^{f}+\Delta tq_{v}\\ {\left(e_{ph0}\right)}_{i,j,k}^{f+1}=\left(1-\Delta t/\tau_{ph}\right){\left(e_{ph0}\right)}_{i,j,k}^{f}+\Delta t/\tau_{ph}\cdot {\left(e_{ph0}^{0}\right)}_{i,j,k}^{f}+\Delta tq_{v}\\ {\left(e_{ph1}\right)}_{i+1,j,k}^{f+1}=\left(1-\Delta t/\tau_{ph}\right){\left(e_{ph1}\right)}_{i,j,k}^{f}+\Delta t/\tau_{ph}\cdot {\left(e_{ph1}^{0}\right)}_{i,j,k}^{f}+\Delta tq_{v}\\ {\left(e_{ph2}\right)}_{i-1,j,k}^{f+1}=\left(1-\Delta t/\tau_{ph}\right){\left(e_{ph2}\right)}_{i,j,k}^{f}+\Delta t/\tau_{r}\cdot {\left(e_{ph2}^{0}\right)}_{i,j,k}^{f}+\Delta tq_{v}\\ {\left(e_{ph3}\right)}_{i,j+1,k}^{f+1}=\left(1-\Delta t/\tau_{ph}\right){\left(e_{ph3}\right)}_{i,j,k}^{f}+\Delta t/\tau_{ph}\cdot {\left(e_{ph3}^{0}\right)}_{i,j,k}^{f}+\Delta tq_{v}\\ {\left(e_{ph4}\right)}_{i,j-1,k}^{f+1}=\left(1-\Delta t/\tau_{ph}\right){\left(e_{ph4}\right)}_{i,j,k}^{f}+\Delta t/\tau_{ph}\cdot {\left(e_{ph4}^{0}\right)}_{i,j,k}^{f}+\Delta tq_{v}\\ {\left(e_{ph5}\right)}_{i,j,k+1}^{f+1}=\left(1-\Delta t/\tau_{ph}\right){\left(e_{ph5}\right)}_{i,j,k}^{f}+\Delta t/\tau_{ph}\cdot {\left(e_{ph5}^{0}\right)}_{i,j,k}^{f}+\Delta tq_{v}\\ {\left(e_{ph6}\right)}_{i,j,k-1}^{f+1}=\left(1-\Delta t/\tau_{ph}\right){\left(e_{ph6}\right)}_{i,j,k}^{f}+\Delta t/\tau_{ph}\cdot {\left(e_{ph6}^{0}\right)}_{i,j,k}^{f}+\Delta tq_{v} \end{array}\right.$$

Moreover, this system of equations must be supplemented with the following initial condition:

(14) $$t=0:\;\;\;\;\;e_{e}(x,y,z,0)=e_{e}(T_{0}^{e}),\;\;\;\;\;e_{ph}(x,y,z,0)=e_{ph}(T_{0}^{ph})$$

and boundary conditions of the form:

(15) $$\left\{\begin{array}{l} x=0,\;0\leq y\leq L,\;0\leq z\leq L:\;e_{e}(0,y,z,t)=e_{e}\left(T_{b1}^{e}\right)\\ x=L,\;0\leq y\leq L,\;0\leq z\leq L:\;e_{e}(L,y,z,t)=e_{e}\left(T_{b2}^{e}\right)\\ y=0,\;0<x<L,\;0\leq z\leq L:\;q_{b3}^{e}(x,0,z,t)=v_{e}\left[e_{e}{\left(T_{e}\right)}_{i,0,k}-e_{e}{\left(T_{e}\right)}_{i,1,k}\right]\\ y=L,\;0<x<L,\;0\leq z\leq L:\;q_{b4}^{e}(x,L,z,t)=v_{e}\left[e_{e}{\left(T_{e}\right)}_{i,m-1,k}-e_{e}{\left(T_{e}\right)}_{i,m,k}\right]\\ z=0,\;0<x<L,\;0\leq y\leq L:\;e_{e}(x,y,0,t)=e_{e}\left(T_{b5}^{e}\right)\\ z=L,\;0<x<L,\;0\leq y\leq L:\;e_{e}(x,y,L,t)=e_{e}\left(T_{b6}^{e}\right)\\ x=0,\;0\leq y\leq L,\;0\leq z\leq L:\;e_{ph}(0,y,z,t)=e_{e}\left(T_{b1}^{e}\right)\\ x=L,\;0\leq y\leq L,\;0\leq z\leq L:\;e_{ph}(L,y,z,t)=e_{e}\left(T_{b2}^{e}\right)\\ y=0,\;0<x<L,\;0\leq z\leq L:\;q_{b3}^{ph}(x,0,z,t)=v_{ph}\left[e_{ph}{\left(T_{e}\right)}_{i,0,k}-e_{ph}{\left(T_{e}\right)}_{i,1,k}\right]\\ y=L,\;0<x<L,\;0\leq z\leq L:\;q_{b4}^{ph}(x,L,z,t)=v_{ph}\left[e_{ph}{\left(T_{e}\right)}_{i,m-1,k}-e_{ph}{\left(T_{e}\right)}_{i,m,k}\right]\\ z=0,\;0<x<L,\;0\leq y\leq L:\;e_{e}(x,y,0,t)=e_{e}\left(T_{b5}^{e}\right)\\ z=L,\;0<x<L,\;0\leq y\leq L:\;e_{e}(x,y,L,t)=e_{e}\left(T_{b6}^{e}\right) \end{array}\right.$$

After determining the energy densities of the electrons and phonons for all seven individual directions, the total energy density at the lattice node can be computed as

(16) $${\left(e_{e}\right)}_{i,j,k}^{f+1}=\sum_{d=0}^{6}{\left(e_{e\;d}\right)}_{i,j,k}^{f+1}$$

(17) $${\left(e_{ph}\right)}_{i,j,k}^{f+1}=\sum_{d=0}^{6}{\left(e_{ph\;d}\right)}_{i,j,k}^{f+1}$$

The temperature at the lattice nodes is then determined using the transformed relations (3) and (4).

**Figure 4:**
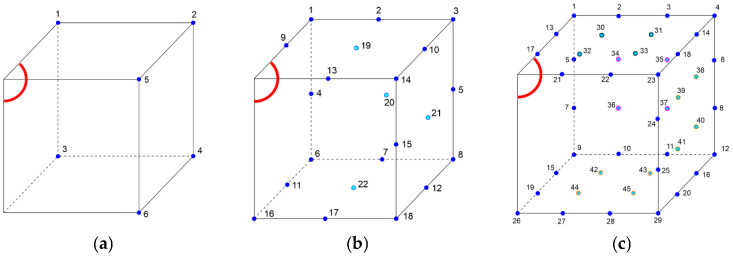
Sensor configurations (the red color indicates the point of laser impact, the dark blue dots indicate the sensors on the edges, and the light blue dots indicate the sensors on the surfaces of the model): (**a**) 6, (**b**) 22, and (**c**) 45 sensors.

The identification tasks were performed for different numbers of boundary sensor points located on the external edges of the cube. Three different cases were considered: 6, 22, and 45 sensors. Figure 4 shows the location of the sensors for each case.

## 3. Identification Algorithms

Identification problems belong to ill-posed problems and can lead to ambiguity in the solution. Moreover, in many cases, the identification function may have a strong multimodal character. Solving this type of problem in practice often involves, on the one hand, the sometimes necessary regularization of the function and, on the other hand, the use of methods resistant to being stuck in a local minimum. For this reason, this work uses an evolutionary algorithm, the Monte Carlo method, and the Nelder-Mead algorithm with a multistart strategy, which belongs to global optimization methods. The various types of these algorithms are described in more detail below.

**Figure 5:**
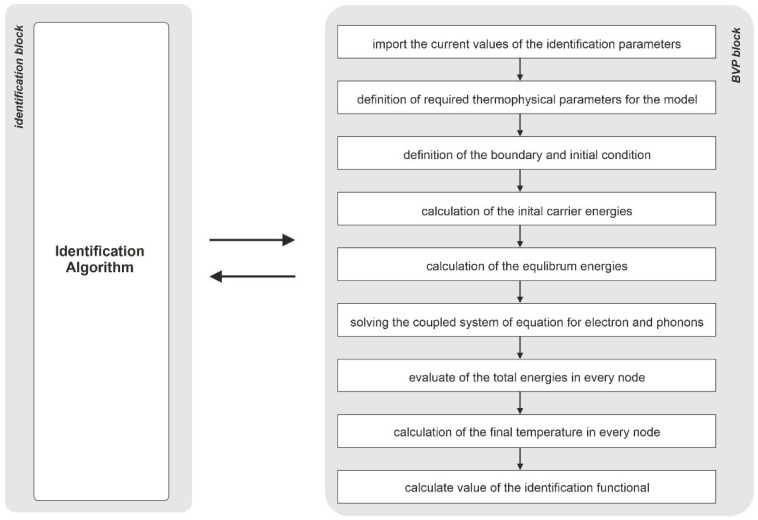
Block diagram of the identification problem.

The coupling between a particular algorithm and the block responsible for solving the overall boundary-value problem (BVP) and calculating the identification function is shown by the diagram in Figure 5. For the current set of identification parameters, a numerical model is created for which the transient heat flow problem is solved by means of the BTE and LBM. Based on the obtained temperature field in space and time, the value of the identification function is determined and sent back to the particular identification algorithm. For the purposes of communication between individual blocks, appropriate interfaces were developed to enable data transfer.

### 3.1. Evolutionary Algorithm

**Figure 6:**
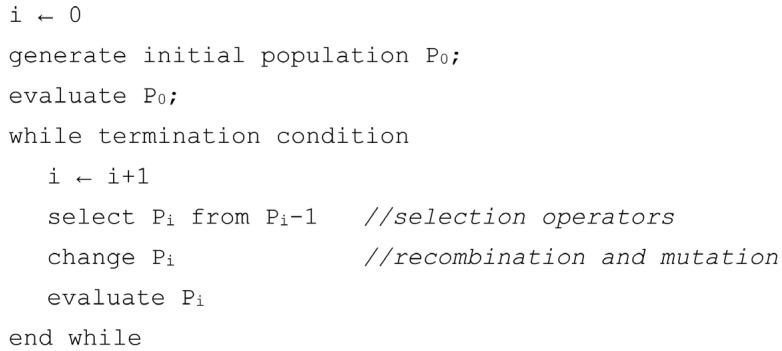
Pseudocode of the EA.

Evolutionary algorithms are algorithms that search for a set of solutions and are established in analogy to the biological evolution of species [28]. Similar to biology, the term “individual” is used to represent a single solution. Evolutionary algorithms (EAs) operate on populations of individuals so, while an algorithm operates, we continually deal with a set of problem solutions. An individual consists of chromosomes. The pseudocode of EA is shown in Figure 6. In the first step, an initial population of individuals is created. The algorithm works iteratively, processing a population of solutions in each iteration. In the present work, the in-house implementation of EA was used and adopted to address the considered problem. The initial population is generated randomly. The two forms of crossover and mutation operators were used: uniform mutation, Gaussian mutation, simple crossover, and arithmetic crossover.

Algorithms operate while a stop condition is unfulfilled, where the maximal number of generations is assumed. Our implementation of the algorithm was tested on several mathematical benchmarks that demonstrated high effectiveness [39]. The algorithm was used for solving many practical problems in the mechanical engineering discipline, e.g., identification of thermal properties of hardening concrete [40], identification of voids and cracks [39], and identification of multiscale problems [41,42].

### 3.2. Nelder-Mead Algorithm

The Nelder-Mead algorithm is often referred to as the creeping simplex method [43]. The principle of the method is based on the construction of an n-dimensional simplex with n + 1 vertices in space. This simplex should be defined in such a way that it can be inscribed on the surface, representing the objective function. For example, a two-dimensional simplex is a triangle. In general, an n-dimensional simplex with n + 1 vertices is a polyhedron spanned by n + 1 basis vectors. The advantage of the method is that the values of the derivatives of the objective function are not needed; however, the convergence of the method is affected by the choice of the initial simplex.

**Figure 7:**
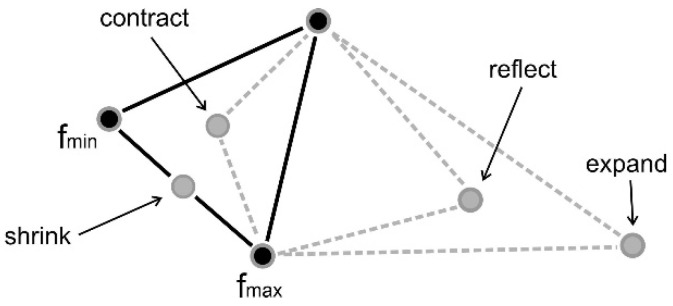
Possible operations in the Nelder-Mead algorithm.

The algorithm begins by calculating the coordinates of the vertex points of the simplex and determining the coefficients of shrinking, contraction, reflection, and expansion operators (ρ, δ, α, and γ). A certain minimum distance between these vertices is required. In successive iterations, the simplex of a n + 1 basis vector is transformed until the termination condition is satisfied (defined as the maximum number of iterations or achieving the minimum with the specified accuracy). Transformations are performed after finding the worst (W), best (B), and second worst (G) points; comparing these points and, given certain rules, the appropriate modification (shrinking, contraction, reflection, or expansion) is then undertaken (Figure 7).

**Figure 8:**
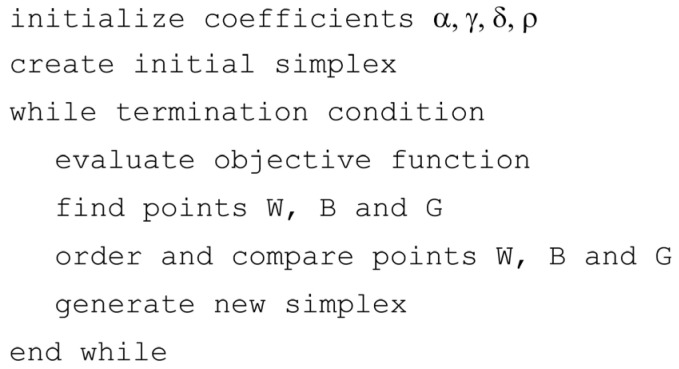
Pseudocode of the Nelder-Mead algorithm.

The simplified pseudocode of the Nelder-Mead algorithm is shown in Figure 8. An implementation of the algorithm, developed in C, was adapted for the purposes of this study [44]. As for the EA, the proper interfaces between the algorithm, the numerical simulations, and the calculation of the identification function were developed.

### 3.3. Monte Carlo Method

Monte Carlo optimization methods can be useful for solving problems with many local optima and complicated constraints [45]. Many different variants of stochastic optimization have been developed in the past. Some of them mimic the classical gradient descent methods by replacing a deterministic gradient with a random approximation (e.g., Robbins-Monro or Kiefer-Wolfowitz algorithm) [46]. The most popular algorithm belonging to Monte Carlo methods is the simulated annealing algorithm.

**Figure 9:**
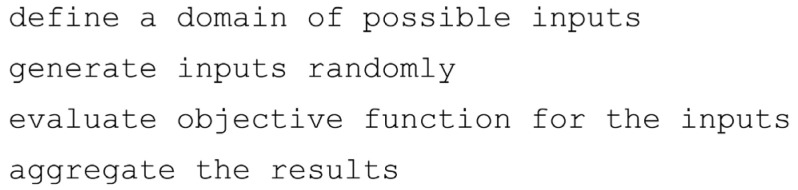
Pseudocode of the Monte Carlo algorithm.

This paper uses the simplest version of this algorithm in which, after defining the searching domain, the solutions are generated randomly with a uniform distribution function. Then, for every solution, the objective function is calculated. The last step is the aggregation of the results and the selection of the optimal outcome. The simplified pseudocode of such an algorithm is shown in Figure 9.

## 4. Results of the Identification Tasks

### 4.1. Identification for Unnoisy Data

The identification task formulated in Section 2.2 was solved for both variants (simultaneous identification of *r_l_* and β and simultaneous identification of *r_l_* and *P*) and different numbers of sensors (6, 22, and 45). The cube of dimensions (a quarter of the model) of 500 × 500 × 500 nm was analyzed. The measured (postulated) temperature field was calculated for the following values of the laser pulse: laser pulse power *P* = 0.9 W, laser beam absorptivity β = 0.4, and radius of the laser beam *r_l_* = 100 nm. The laser pulse modeled as the heat source component is located at one of the edges of the model (Figure 2), whereas zero heat flux was applied to all sides of the cube (the adiabatic boundary condition). The rest of the parameters for the numerical model were collected in Table 1.

The box constraints imposed on the identification parameters for all the variants and cases belong to the ranges:

(18) $$\begin{array}{c} 50\;\mathrm{nm}\;\leq \;r_{l}\leq 150\;\mathrm{nm},\\ 0.1\leq \beta \leq 1.0,\\ 0.1\;W\leq P\leq 2.5\;W. \end{array}$$

After some preliminary calculations, identification by means of EA was performed for the following values of evolutionary operators: a population size of 100, the number of generations at 500, the probability of simple crossover at 0.1, the probability of the arithmetic crossover at 0.1, the probability of the Gaussian mutation at 0.7, the range factor of the Gaussian mutation at 0.9, and the probability of the uniform mutation at 0.1. Identification tasks were also carried out for different values of the algorithm parameters. The probabilities of the simple and arithmetic crossover were increased and decreased at the expense of the Gaussian mutation, with no improvement in the identification results.

The algorithm was run 30 times with a randomly generated starting population. The results of the identification were aggregated. Table 2 contains all the results of the identification for the two variants of identification and the three considered cases. For each variant and identification case, the relative errors (ER) for the identified parameters were calculated. Based on the results from all 30 runs of the algorithm, the mean error value was calculated (penultimate column in the table, MR). Taking into account the calculated relative errors (ER) for identified parameters, the best, worst, and median tests were chosen, as shown, for example, in Table 3. Since, for each identification case, the error was calculated for a pair of parameters, the best solution, worst solution, and median were determined via the summed error of these parameters (the last column of Table 3). In addition, it was assumed that a single identification task can be considered acceptable if the error of the solution for each of the identified parameters is less than 5%. This information can be found in the last column of Table 2.

Analyzing the obtained results, it can be seen that only a few (in one case, not at all) of the 30 independent runs of the algorithm can be considered acceptable. As mentioned earlier, identification tasks were also solved using the Nelder-Mead algorithm and the Monte-Carlo method. To make the comparison meaningful, the same number of function evaluations was assumed for each identification task (equal to 50,000). For the Nelder-Mead algorithm, randomly initialized starting points were equal to 1000 and, for each point, the maximal number of iterations was 50. The values of the coefficients of reflection, expansion, contraction, and shrinking operators (α, γ, δ, and ρ) were set at 1, 2, 0.5, and 0.5, respectively. In addition, the algorithm sets the value of tolerance on the simplex solutions’ coordinates and tolerance on the function value at 0.001. For the Monte-Carlo method, 50,000 solution points were generated randomly with a uniform distribution function. Based on the calculations using the same method as for the EA, the errors and statistical parameters were calculated. Table 4 and Table 5 contain results for the identification using the Nelder-Mead algorithm and the Monte-Carlo method, respectively.

**Figure 10:**
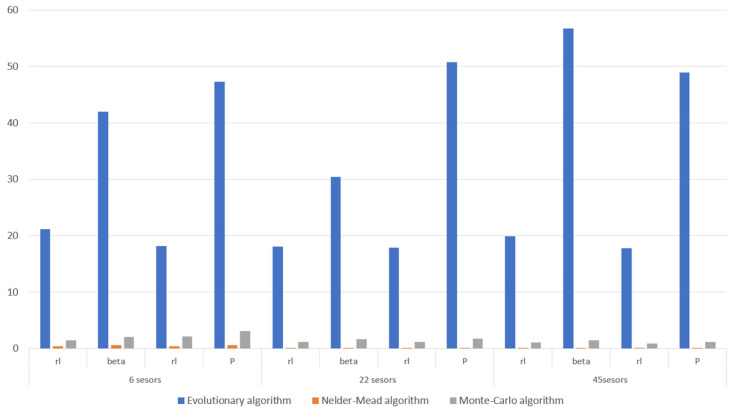
Comparison of the mean identification errors for all the considered variants and cases.

Based on the penultimate column in Table 2, Table 4 and Table 5, a bar chart was prepared to compare the mean error for all the variants and cases. Figure 10 presents such a comparison.

### 4.2. Identification for Data with Noise

As mentioned earlier, with the currently available technical instrumentation, it is impossible to make realistic measurements in such an extreme temporal and spatial regime. Despite this, it was decided to also test the effectiveness of the applied algorithms for data considering measurement uncertainty. In this case, the sensor temperature measurement data, denoted as ${\hat{T}}_{ij}$ in Equation (10), considers noise and includes uncertainty at three different levels. The Gaussian distribution density function is used to generate noisy data. It was assumed that the expected value of the temperature is equal to the deterministic value, while the standard deviation is equal to 1/3 of the maximal error. A more detailed explanation of the consideration of measurement uncertainty can be found in Ref. [39]. In this work, it was assumed that the maximum measurement error was either 2%, 5%, or 10%. Computations were carried out using all the developed and applied algorithms for the case of the simultaneous identification of *r*_l_ and *β* parameters (variant 1) and the six measurement points. All the parameters of the numerical model, as well as the parameters used in the optimization algorithms, are the same as those used for the identification without noise.

**Figure 11:**
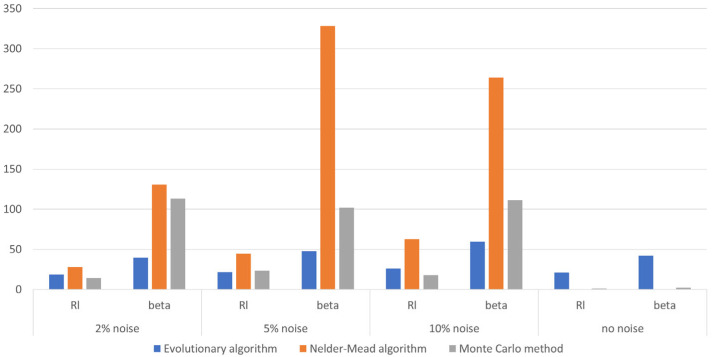
Comparison of the mean identification errors for different levels of noise in the data.

The identification results were aggregated and, similar to the previous subsection, compared by selecting the best, worst, and median case. Table 6, Table 7 and Table 8 contain results for the identification with different levels of noise. The same as in the previous subsection, the bar chart presented in Figure 11 was prepared for the mean error, which was calculated after 30 independent runs of each algorithm. In this figure, the results of the identification without noisy data are also pointed out for comparison. Analyzing the obtained results, it can be seen that the sum of the mean error of the solution obtained by the evolutionary algorithm slightly increases as the noise level increases. Assuming an identical criterion for a successful identification task (as in Section 4.1) with 2% and 5% noise, slightly better results were obtained compared with the solution without noise. However, by analyzing the value of the mean error (the penultimate column), it can be concluded that the results obtained are very similar. The use of the other two algorithms in this case was discovered to be completely ineffective, which is evident in both the data in Table 6, Table 7 and Table 8 and Figure 11.

A more detailed discussion of the obtained results and an attempt to explain them are included in the next section.

## 5. Discussion

**Figure 12:**
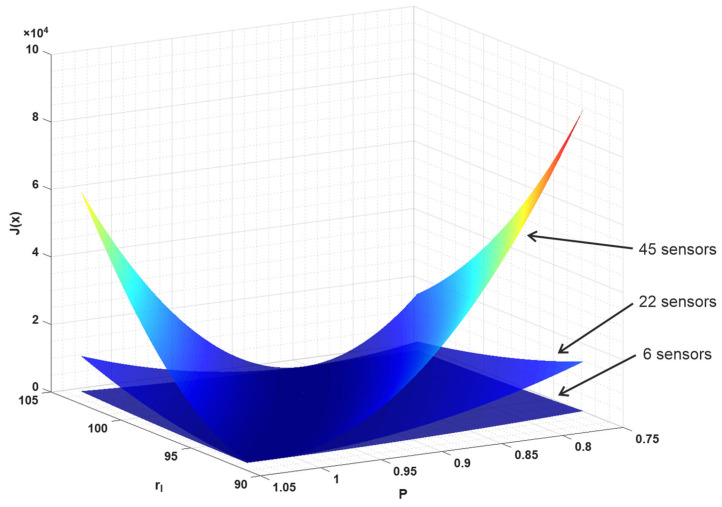
Graph of the identification function for a different number of sensors.

Analyzing the identification for the ideal deterministic (i.e., with no noise) obtained results that show that the accuracy is particularly poor for the EA, but quite good with the Monte-Carlo method and best with the Nelder Mead algorithm. One would expect just the opposite results due to the distinctive characteristics of the three algorithms. Our implementation of the evolutionary algorithm used in this article, like other implementations and other versions of metaheuristic algorithms, has been successfully used in the past to solve optimization and identification problems, where the physical problem was simulated using the finite element method, the boundary element method, and the finite difference method [39,47,48]. It is common knowledge that each of these numerical methods has resulted in an objective function that is no longer smooth. The nature of these disturbances and the size of the resulting “peaks” depend on both the method and the size of the mesh assumed. It is found that, in the physical problem considered in this paper, the perturbations causing the non-smooth objective function are quite different, which causes the problem of obtaining a satisfactory solution with EA. Interestingly, the Boltzmann method used, i.e., the interval version for the 1D problem used to identify laser parameters using EA, gave satisfactory results [24]. Uncertainties most likely cause the objective function to be “smoothed out.” The evolutionary algorithm is generally resistant to convergence at local minima, while the convergence of the Nelder-Mead algorithm strongly depends on the starting point adopted. In order to examine the reasons for this situation more closely, a graph of the identification function was prepared. Figure 12 presents a graph of the identification function for the identification parameters *r_l_* and *P* (variant 2), which considers a different number of sensors.

**Figure 13:**
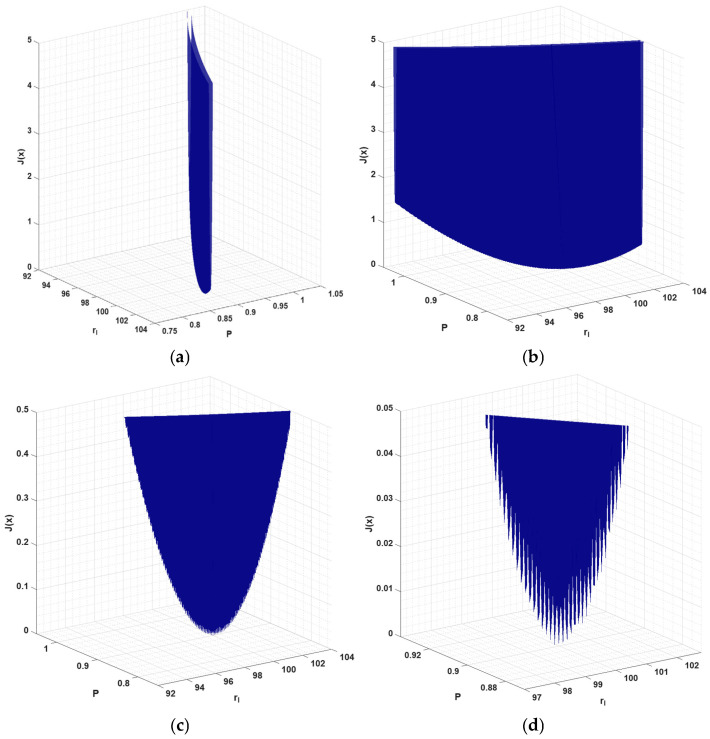
Enlarged graphs of the landscape of the identification function near the global minimum: (**a**), (**b**) for a range of 0 to 5, (**c**) 0 to 0.5, and (**d**) 0 to 0.05 objective function values.

As can be seen, increasing the number of sensors enhances the slope of the function graph. This relationship and a similar shape for the graph of the function is also observed for variant 1 (identification parameters *r_l_* and β). Note, however, that this is not a graph of the function for the entire domain under consideration. For clarity, the range of variables has been limited here to the intervals $92\;\mathrm{nm}\;\leq \;r_{l}\leq 104\;\mathrm{nm}$ and 0.75 W≤P≤1.05 W. The global trajectory of the objective function has a smooth form, but, if we examine the magnified area of the global minimum, we will find that a high number of local minima (located throughout the valley) can be found. Such magnification is shown in Figure 13. It should be noted that a narrow, almost flat valley extends across the entire domain. Figure 13a,b show only part of the graph of the function from different perspectives. Figure 13c,d display enlarged areas where the value of the objective function on the vertical axis is limited to 0.5 and 0.05, respectively.

An experiment to change the probabilities of the evolutionary operators (described in Section 4) was not able to improve the identification results. The vast number of very narrow peaks of local minima results in a lack of effective EA performance. At the final stage of the evolution, all the individuals in the population are distributed throughout the entire valley. However, very small differences in the value of the objective function do not direct the search area toward the location of the global optimum. This is most likely the main reason for such poor EA performance.

The optimal solutions obtained with the EA are of comparable quality with respect to the other algorithms (Table 2). However, acceptable solutions were found in only a minority of cases. For such a number of calls of the objective function (i.e., the population size and the number of generations), it can be concluded that, out of 30 independent runs, at least one provides a solution at the error level of 5%. Previous mentions of the performance of EA are formulated mainly on the basis of the obtained mean and median error.

On the other hand, a surprisingly good performance was obtained with the Nelder-Mead algorithm, in which the solution obtained generally depends strongly on the starting point taken. By observing the improvement in the value of the identification function in successive iterations of the algorithm, for completely different starting points, the correct determination of the search direction was observed. This means that sharp peaks are somehow invisible to the creeping simplex method, even for default parameter values. This arises due to the case where the algorithm searched as if it were a smooth function that has no complex shape (Figure 12). Moreover, analyzing the results collected in Table 2, Table 4 and Table 5, it can be seen that increasing the number of sensors improves identification accuracy. This is a fairly typical phenomenon that is also observed for many other identification tasks. This relationship is not always observed for the EA, mainly for the reasons mentioned earlier.

The results for the noisy temperature measurement data were analyzed only for the first variant with six measurement points. The mean identification error slightly increases as the noise level increases. However, as for the identification without noise, it is difficult to consider the results as satisfactory when only a few runs of the algorithm obtain acceptable results. The identification results for the individual runs of the algorithm are summarized in Table 6, Table 7 and Table 8, which include data for the smallest value of the identification function (10). It is determined that, for a slightly worse value of the objective function, a more accurate solution could be obtained in many cases. In this case, the identification process would have to be stopped a little earlier. This is not demonstrated in the data presented in this work, as the full course of the changes in the objective function in successive iterations of the algorithm is not included (but only in the final result). This is, of course, due to the fact that the landscape of the identified function for noisy data is different compared with data without noise, and the minimum of the function is not found for the same parameter value as for data without noise.

An attempt to improve the obtained solution by stopping the identification algorithm earlier is a different issue not considered in this paper. This problem is known and described in the literature regarding identification in cases of uncertain measurement data and is very similar to Morozov’s discrepancy principle [49,50].

A different matter that could be discussed in this case is the assumption that a very large number of minima can be reduced by changing the mesh size in the LMB method; this is unfortunately not possible in practical applications and numerical implementations. Such modification in the method (using the D3Q7 model) is limited due to the fact that the lattice step should have dimensions comparable with the mean free path for electron-phonon scattering [51,52,53]. Additional research into the approach of adding a regularizing term to the objective function also seems to be justified. However, the regularization effect must be chosen in an appropriate way to avoid the danger of losing important information about the objective function with too much regularization influence. This is certainly worthy of future research.

Errors for the obtained solutions with the Nelder-Mead algorithm reach up to several hundred percent, which was found to be the worst-case scenario in comparison with the identification with no noise. The “modified landscape” of the objective function, in this case, disables such algorithms from obtaining a satisfactory solution regardless of the choice of the starting point of the algorithm. It may come as little surprise that a completely random search (i.e., the Monte-Carlo method), in this case, gives better results. The individual identification tasks presented in the paper can be treated as time-consuming. In individual cases, of course, it would be possible to reduce the total time. The number of 50,000 objective function calls was chosen arbitrarily, mainly to allow a fair comparison of the algorithms used in the work. A single boundary-value problem for the developed LBM numerical model, despite algorithmic complexity of the order of (O(n^2^)), did not require a long simulation time (less than 1 s on a personal computer). For more complex numerical models, a different approach would be required, such as using a metamodel of the boundary-value problem or the use of parallel calculations. The problem of increasing the efficiency of the identification process, although computationally significant, was not the main topic of this paper.

## 6. Concluding Remarks

This paper presents a method for solving the inverse task of determining the ultra-short laser parameters for a 3D model of a thin metal film. The direct problem is solved using the LBM and, for the numerical implementation, in-house numerical code is developed by utilizing the D3Q7 model. An identification function using a different number of temperature sensors at the edges of the numerical model was proposed and numerically implemented. Although it is currently impossible to carry out a real experiment in such an extremely small temporal and spatial regime, this research provided interesting insights into the physical nature of the identification problem under consideration. The main findings of the present study can be summarized as follows:

- The common feature of evolutionary algorithms, which is to assure an appropriate balance between exploration and exploitation of the feasible solution space, unfortunately did not yield satisfactory results in this case. The reason for this is the very high density of peaks with local minima, which is significantly higher than when using other numerical methods, such as FEM, BEM or FDM.
- Good and satisfactory results were obtained using the Nelder-Mead algorithm, in which the solution obtained usually depends on the selected starting point. In this case, sharp peaks did not prevent a satisfactory solution from being obtained for the creeping simplex method. The correct search direction was observed even for completely different starting points.
- Interesting results were also obtained for measurement data that included Gaussian noise at different levels. The most resilient algorithm for noisy data was determined to be the evolutionary algorithm. It seems that the results in this case could be improved by using the previously mentioned Morozov’s discrepancy principle. The implementation of this principle to such a metaheuristic algorithm requires further research and is also worth considering.
- A remarkable phenomenon observed concerning the unusual nature of the identification function, unusual for numerical methods, can be additionally used in another way. The problem presented in this paper can also certainly be used as a test function (i.e., a benchmark) to evaluate the quality of optimization algorithms. Despite the fact that the use of the Monte Carlo method and Nelder-Mead algorithm with a multi-start allowed us to obtain significantly better results compared with EA (for data with no noise), none of the optimization algorithms should be used without a deeper study of a problem such as this one. For a small number of parameters, it is possible to visualize at least a fragment of the identification function domain. However, this should be undertaken with a sufficiently high resolution to circumvent any loss of information relating to local drastic changes in the function. In the authors’ opinion, the identification task for non-noisy data could be a good example of a new test function for other metaheuristic algorithms.

## Figures and Tables

**Table 1:** Parameters for the numerical model.

Parameter	Value
Lattice steps	Δx = Δy = Δz = 50 nm
Time step	Δt = 0.01 ps
Number of time steps	400
Relaxation time for electrons	τ_e_ = 0.04 ps
Relaxation time for phonons	τ_ph_ = 0.8 ps
Debye temperature	Q_D_ = 170 K
Fermi energy	ε_F_ = 5.53 eV
Coupling coefficient	G = 2.3 × 10^16^ W/m^3^ K
Boltzmann constant	k_b_ = 1.38065 × 10^−23^ J/K
Initial temperature	T_0_ = 300 K

**Table 2:** Results of the identification by means of the evolutionary algorithm.

		Best		Worst		Median		MR	ER < 5%
		Value	ER	Value	ER	Value	ER		
6 sensors	*r_l_*	96.613	3.387	64.423	35.577	124.353	24.353	21.181	0
	β	0.421	5.196	0.979	144.816	0.302	24.393	41.964	
	*r_l_*	99.669	0.331	62.124	37.876	111.921	11.921	18.145	2
	*P*	0.904	0.479	2.471	174.531	0.773	14.118	47.239	
22 sensors	*r_l_*	100.881	0.881	72.133	27.867	123.790	23.790	18.024	3
	β	0.395	1.267	0.727	98.745	0.302	24.482	30.432	
	*r_l_*	100.002	0.002	62.307	37.692	120.726	20.726	17.835	5
	*P*	0.900	0.0002	2.459	173.249	0.701	22.161	50.788	
45 sensors	*r_l_*	103.761	3.761	64.362	35.638	84.806	15.194	19.892	1
	β	0.380	4.885	0.982	145.380	0.519	29.657	56.718	
	*r_l_*	99.361	0.639	62.458	37.542	85.693	14.306	17.773	2
	*P*	0.908	0.901	2.427	169.716	1.146	27.289	48.896	

**Table 3:** Exemplary summary of the identification results (the *r_l_* and β variants) with six sensors.

Test No.	f(x)	r_l_	β	ER(r_l_)	ER(β)	ΣER	
1	−2.156	138.912	0.269	38.912	33.049	71.961	
2	−0.653	64.512	0.975	35.489	143.826	179.315	
3	−0.301	78.540	0.602	21.460	50.429	71.889	
4	−3.491	147.181	0.253	47.181	36.865	84.046	
5	−0.025	94.481	0.435	5.5195	8.797	14.3172	
6	−0.286	79.190	0.592	20.810	47.966	68.776	
7	−0.707	124.353	0.302	24.353	24.393	48.745	← median
…	…	…	…	…	…	…	
24	−0.656	64.423	0.979	35.577	144.815	180.393	← worst
…	…	…	…	…	…	…	
27	−0.01	96.613	0.421	3.387	5.195	8.583	← best
28	−0.587	67.144	0.869	32.856	117.327	150.183	
29	−0.658	123.607	0.304	23.607	23.862	47.468	
30	−0.021	104.709	0.375	4.709	6.237	10.946	

**Table 4:** Results of the identification by means of the Nelder-Mead algorithm.

		Best		Worst		Median		MR	ER < 5%
		Value	ER	Value	ER	Value	ER		
6 sensors	*r_l_*	100.027	0.027	100.704	0.704	99.561	0.439	0.413	30
	β	0.399	0.028	0.396	1.0017	0.402	0.633	0.593	
	*r_l_*	100.068	0.068	100.723	0.723	100.432	0.432	0.417	30
	*P*	0.899	0.096	0.891	1.0271	0.894	0.6248	0.596	
22 sensors	*r_l_*	100.001	0.001	99.884	0.1162	99.951	0.0488	0.0513	30
	β	0.399	0.001	0.401	0.1683	0.4003	0.07	0.0737	
	*r_l_*	99.999	0.0009	100.127	0.127	99.9718	0.0282	0.0418	30
	*P*	0.899	0.0016	0.898	0.1856	0.900	0.043	0.0609	
45 sensors	*r_l_*	100.000	0.0	100.034	0.034	100.018	0.018	0.0173	30
	β	0.3999	0.001	0.3998	0.0475	0.3998	0.0255	0.0242	
	*r_l_*	99.999	0.0004	100.028	0.028	99.985	0.0147	0.0137	30
	*P*	0.900	0.0016	0.8996	0.0398	0.9002	0.0192	0.0194	

**Table 5:** Results of the identification by means of the Monte-Carlo method.

		Best		Worst		Median		MR	ER < 5%
		Value	ER	Value	ER	Value	ER		
6 sensors	*r_l_*	99.933	0.067	103.872	3.872	100.965	0.965	1.422	29
	β	0.400	0.101	0.379	5.208	0.394	1.368	2.008	
	*r_l_*	99.960	0.04	95.176	4.823	97.987	2.012	2.103	26
	*P*	0.900	0.053	0.968	7.556	0.926	2.985	3.064	
22 sensors	*r_l_*	100.093	0.093	97.171	2.829	100.958	0.958	1.159	30
	β	0.399	0.169	0.417	4.284	0.394	1.361	1.677	
	*r_l_*	100.026	0.026	95.754	4.246	99.0337	0.966	1.196	29
	*P*	0.8997	0.03	0.960	6.697	0.9128	1.428	1.756	
45 sensors	*r_l_*	100.043	0.043	96.786	3.214	100.824	0.824	1.050	30
	β	0.400193	0.048	0.4189	4.709	0.396	1.111	1.494	
	*r_l_*	100.039	0.039	97.156	2.844	100.477	0.477	0.866	30
	*P*	0.900322	0.036	0.938	4.177	0.894	0.7101	1.214	

**Table 6:** Results of the identification with noise by means of the evolutionary algorithm.

		Best		Worst		Median		MR	ER < 5%
		Value	ER	Value	ER	Value	ER		
2% noise	*r_l_*	98.695	1.305	65.006	34.994	83.749	16.251	18.648	4
	β	0.412	2.916	0.956	139.024	0.531	32.737	39.668	
5% noise	*r_l_*	101.368	1.368	64.595	35.405	80.345	19.655	21.470	2
	β	0.400	0.012	0.964	141.095	0.579	44.641	47.754	
10% noise	*r_l_*	97.241	2.759	65.707	34.293	140.037	40.037	26.086	1
	β	0.405	1.337	0.935	133.685	0.264	33.984	59.467	

**Table 7:** Results of the identification with noise by means of the Nelder-Mead algorithm.

		Best		Worst		Median		MR	ER < 5%
		Value	ER	Value	ER	Value	ER		
2% noise	*r_l_*	98.892	1.108	50.274	49.726	130.964	30.964	28.112	3
	β	0.405	1.260	2.756	589.078	0.283	29.158	130.818	
5% noise	*r_l_*	116.948	16.948	50.007	49.993	60.491	39.509	44.593	0
	β	0.323	19.187	2.850	612.428	1.180	194.880	328.211	
10% noise	*r_l_*	110.498	10.498	50.086	49.914	207.147	107.147	62.771	0
	β	0.356	11.096	2.958	639.613	0.185	53.766	263.865	

**Table 8:** Results of the identification with noise by means of the Monte-Carlo method.

		Best		Worst		Median		MR	ER < 5%
		Value	ER	Value	ER	Value	ER		
2% noise	*r_l_*	147.995	47.995	92.998	7.002	95.800	4.200	14.084	0
	β	0.562	40.623	0.998	149.515	0.953	138.264	113.369	
5% noise	*r_l_*	145.305	45.305	93.177	6.823	94.049	5.951	23.149	0
	β	0.569	42.134	0.999	149.843	0.975	143.765	102.008	
10% noise	*r_l_*	136.910	36.910	91.142	8.858	92.791	7.209	18.114	0
	β	0.389	2.751	0.997	149.299	0.980	145.085	111.328	

## Data Availability

The original contributions presented in this study are included in the article. Further inquiries can be directed to the corresponding author.

## Abbreviations/Nomenclature

The following nomenclature and abbreviations are used in this manuscript:

**Nomenclature**	
**Roman symbols**	
f	distribution function
v	velocity vector
g	carrier generation coefficient
e	energy density
Q	source function
$Q^{\prime }$	source function associated with laser irradiation
$k_{b}$	Boltzmann constant
$n_{e}$	electron density
ћ	reduced Planck constant
G	coupling coefficient
P	power of the stationary laser pulse
$r_{l}$	radius of the laser beam
J	identification functional
x	vector of the identification parameters
T	computed temperatures in sensors
$\hat{T}$	postulated temperatures in sensors
**Greek symbols**	
β	laser beam absorptivity
$\varepsilon_{F}$	Fermi energy
η	phonon density
τ	relaxation time
Θ_D_	Debye temperature
**Subscripts**	
*e*	electrons
*ph*	phonons
*i*	index of sensor point
*j*	index of time interval
**Superscripts**	
0	in the equilibrium state
**Abbreviations**	
LBM	Lattice Boltzmann Method
BTE	Boltzmann transport equations
DPLE	Dual phase lag equation
D3Q7	Numerical implementation of the three-dimensional model using LBM
BVP	Boundary Value Problem
EA	Evolutionary Algorithm
ER	Relative error
MR	Mean error
